# Supplementary material for: Complexome profiling of the Chlamydomonas psb28 mutant reveals TEF5 as an early PSII assembly factor
Source: Plant Cell. 2025 Mar 17;37(6):koaf055. doi: 10.1093/plcell/koaf055 (PMC12164590; doi:10.1093/plcell/koaf055)
Supplement: koaf055_Supplementary_Data [file koaf055_supplementary_data.zip › psb28_interactive.zip!/plots/intensity/qualityPlots/peptides/TOC75_Cre03.g175200.t1.2.html]

### Cre03.g175200.t1.2

Trivial name: TOC75  
SeqCoverage [median]: 17.70  
Mass: 87.23 kDa  
  
 

| Sequence | MissedCleavage | Mass [Da] | wtIntensity | muIntensity | Score |
| --- | --- | --- | --- | --- | --- |
| ADKIIEASGFQR | 1 | 1333.70 | 389.13 | 0.00 | 114.87 |
| ANVTEAK | 0 | 731.38 | 240.07 | 504.56 | 166.86 |
| APNTVVLHAK | 0 | 1048.60 | 0.00 | 495.90 | 40.50 |
| FINGNQLGER | 0 | 1146.58 | 0.00 | 617.37 | 78.33 |
| FLEAPFLPK | 0 | 1060.60 | 2061.77 | 0.00 | 103.74 |
| FLELATEVR | 0 | 1076.59 | 0.00 | 1436.71 | 67.90 |
| GYSHGEIGAAR | 0 | 1116.53 | 1222.88 | 2188.50 | 86.34 |
| IDNVSVR | 0 | 801.43 | 1242.99 | 6287.42 | 117.10 |
| IIEASGFQR | 0 | 1019.54 | 614.50 | 1620.51 | 72.48 |
| KAPNTVVLHAK | 1 | 1176.70 | 2301.64 | 3924.26 | 111.63 |
| KLGLSGGIYNR | 1 | 1176.66 | 0.00 | 1411.91 | 59.42 |
| KTSTIFTPGGESEVPPVFVDR | 1 | 2262.15 | 0.00 | 1888.41 | 40.01 |
| LTTEQLWK | 0 | 1017.55 | 339.03 | 1362.50 | 62.66 |
| NAQLFNTR | 0 | 962.49 | 529.61 | 0.00 | 65.04 |
| NRIEQWYQSR | 1 | 1378.67 | 0.00 | 395.37 | 39.15 |
| TEYVDGK | 0 | 810.38 | 658.65 | 1031.50 | 99.00 |
|  |  |  | 9600.3 | 23164.9 |  |
